# Recurrence following invasive GAS infections in adults: Triumph of virulence or failure of immunity?

**DOI:** 10.1080/21505594.2025.2563765

**Published:** 2025-09-29

**Authors:** Marek Stefan, Marie Brajerova, Suhanya Prasad, Eliska Bebrova, Jan Berousek, Jiri Pozniak, Silvia Jarosciakova, Andrea Rennerova, Tomas Milota, Otakar Nyc, Pavel Drevinek, Marcela Krutova, Milan Trojanek

**Affiliations:** a Department of Infectious Diseases and Travel Medicine, Second Faculty of Medicine, Charles University and Motol University Hospital, Prague, Czech Republic; b Department of Medical Microbiology, Second Faculty of Medicine, Charles University and Motol University Hospital, Prague, Czech Republic; c Department of Anaesthesiology and Intensive Care, Second Faculty of Medicine, Charles University and Motol University Hospital, Prague, Czech Republic; d Third Department of Surgery, First Faculty of Medicine, Charles University and Motol University Hospital, Prague, Czech Republic; e Childhood Leukaemia Investigation Prague, Department of Paediatric Haematology and Oncology, Second Faculty of Medicine, Charles University and Motol University Hospital, Prague, Czech Republic; f Department of Immunology, Second Faculty of Medicine, Charles University and Motol University Hospital, Prague, Czech Republic

**Keywords:** Streptococcus pyogenes, necrotizing fasciitis, sepsis, cellulitis, lectin pathway complement deficiency, CD19+ deficiency

## Abstract

Since late 2022, an increase in *Streptococcus pyogenes* (Group A *Streptococcus*, GAS) infections, both non-invasive and invasive (iGAS), has been reported globally. This study investigates iGAS cases complicated by recurrent infection (rGAS). From January to September 2023, four adults with severe iGAS suffered from rGAS. Clinical and whole-genome sequencing data were analysed. All patients required ICU admission and surgical debridement during their initial iGAS. The median interval between the initial iGAS and rGAS was 25.5 days, with a median duration of antibiotic treatment of 25 and 17.5 days, respectively. Patients A (female, age 69) and B (male, age 46) had upper limb necrotising fasciitis complicated by a subsequent cellulitis at the exact location. GAS *emm*1.3 (M1_UK_) was isolated in both patients, but patient A´s isolates carried a type-IV secretion system (T4SS), and this patient had a more severe course of infection. Patient C (male, age 66) had two episodes of bacteremia caused by GAS *emm*89.0 carrying T4SS and GAS *emm*12.37 with a frameshift in the *rocA* gene. Patient D (female, age 69) had upper limb cellulitis with bacteremia during the initial iGAS and upper limb cellulitis with septic gonitis as two concurrent manifestations of rGAS. All three isolates were identical, belonging to *emm*12.0 and carrying a 79 amino acid deletion in the SclA. Patients B and C had a reduced function of the complement lectin pathway and CD19+ lymphocyte deficiency. A combination of strain virulence factors and host immune deficiencies may predispose patients with iGAS to recurrence.

## Introduction

Since the end of 2022 and throughout 2023, there has been a global increase in reported Group A *Streptococcus* (GAS) infections, along with an upsurge of invasive streptococcal infections (iGAS) [1–8]. This trend is primarily attributed to the spread of the recently identified hypervirulent *S. pyogenes* M1_UK_ lineage [9,10], combined with reduced exposure to airborne pathogens during COVID-19 lockdowns, which likely led to a decline in population-wide immune system stimulation.

M1_UK_ is characterised by 27 single-nucleotide polymorphisms (SNPs) in its core genome and is known for producing elevated levels of pyrogenic exotoxin A (SpeA). However, this alone does not fully explain its success, as other lineages, such as M1_23SNPs_, are also hyperproducers of SpeA but have not achieved similar prevalence [11].

While GAS *emm*1 is the most commonly isolated strain from iGAS cases in industrialised nations, other *emm* types, such as *emm*3, *emm*12, *emm*49 and *emm*89 are also frequently linked to iGAS [12,13]. Thus, additional mechanisms may contribute to GAS virulence, including spontaneous mutations in virulence genes or horizontal gene transfer *via* integrative and conjugative elements (ICEs). ICEs are mobile genetic elements integrated into the bacterial chromosome and spread through conjugation. Their core structure encodes genes for recombination, conjugative transfer (e.g. type 4 secretion system, T4SS), and regulation [14]. Furthermore, ICE can carry accessory genes that confer selective advantages, such as antibiotic resistance and increased pathogenicity.

iGAS is defined as a clinical illness with the isolation of GAS from a sterile body site [15]. However, it encompasses a diverse range of infections with varying clinical severity. Conditions like toxic shock syndrome, sepsis, necrotising fasciitis and lower respiratory tract infections are associated with higher mortality rates compared to other iGAS manifestations, such as deep cellulitis, upper respiratory tract infections, and osteoarticular infections [15,16].

Management of iGAS typically involves antibiotic therapy, primarily penicillin G or other antistreptococcal beta-lactams combined with protein synthesis inhibitors like clindamycin to suppress production of streptococcal toxins. Treatment may also include surgical debridement, intensive care, and, in some cases, the administration of intravenous immunoglobulins (IVIG) [17,18]. However, there are currently no international guidelines for the treatment of iGAS.

GAS is well-documented for causing recurrent infections, including erysipelas/cellulitis, tonsillopharyngitis and vulvovaginitis [19–21]. Conversely, recurrent GAS infections complicating iGAS cases are relatively rare in the literature [22–24].

Since late 2022 and throughout 2023, our hospital has observed atypical iGAS cases marked by poor response to seemingly adequate therapy. This study explores four prolonged cases of severe iGAS, complicated by either noninvasive or invasive recurrent infections (rGAS), and provides a detailed analysis of the causative *S. pyogenes* strains.

## Material and methods

### Patient selection

From 25 January - to 30 September 2023, *S. pyogenes* isolates from inpatients and outpatients at Motol University Hospital in Prague, Czech Republic, were collected and stored at −80°C using a cryobank system (ITEST, Czech Republic). Clinical symptoms of patients with GAS positivity confirmed through culture or 16S rDNA detection [25] were retrospectively reviewed. Patients with severe iGAS that manifested with a recurrent GAS infection (rGAS), as defined below, were selected for further analysis. For these patients, demographic data, clinical courses, laboratory findings, microbiological and imaging results, antibiotic regimens and other therapeutic interventions were retrospectively collected from medical records. Written consent for photography and publication of the case report was obtained from all patients.

### Definition of severe iGAS

Severe invasive *S. pyogenes* infection (iGAS) was defined as a severe clinical illness meeting at least one of the following criteria: a) GAS bloodstream infection, irrespective of the primary site of infection, or b) severe skin and soft tissue infections (e.g. necrotising fasciitis and/or myositis) with GAS confirmed by culture or PCR from the infected site, with or without blood culture positivity, or c) lower respiratory tract infections (e.g. pneumonia/mediastinitis or empyema) with GAS confirmed by culture or PCR from the infected site, with or without blood culture positivity [16].

Toxic shock syndrome (TSS) was assessed using its original definition [26].

Recurrent GAS infection was defined as a new episode of infection occurring within six months of the initial iGAS episode, characterised by new clinical and laboratory signs of infection and confirmed by positive culture or PCR for GAS from the infected site.

### Immunology testing

The patients with rGAS underwent immunology testing during the follow-up phase. Initial screening included lymphocyte subpopulation analysis using flow cytometry, measurement of immunoglobulin IgG, IgM and IgA levels, C3 and C4 levels, and evaluation of classical, alternative, and lectin complement pathway activities using ELISA (Wieslab Complement system green Classical, alternative, MBL pathways, SVAR life science AB, Sweden). Further, genetic testing using a panel encompassing 658 genes (SureSelect XT HS2 library preparation kit, Agilent, USA) sequenced on NextSeq 2000 (Illumina, USA) associated with immune and hematopoietic disorders was performed (Supplementary Table S1).

### *Characterisation of causative* S. pyogenes *isolates*

Identification of GAS isolates was performed using matrix-assisted laser desorption/ionisation time-of-flight (MALDI -TOF) mass spectrometry (Biotyper v 3.1, Bruker Daltonics, Germany).

Antimicrobial susceptibility testing was performed for 12 antimicrobials using the microdilution method with the MIC G+ suspension medium (Erba Lachema, Germany) and interpreted according to the EUCAST breakpoints (v14.0), except for gentamicin and chloramphenicol, for which susceptibility categorisation breakpoints are not available. Whole-genome sequencing (WGS) was performed on GAS isolates from both initial infections and recurrences. DNA extraction was performed after thawing the isolates on blood agar (Oxoid, UK), followed by overnight culture at 37°C with 5% CO_2._ Subculturing was then performed on chocolate blood agar (Oxoid, UK) after a 48-hour culture under the same conditions. DNA was purified using the MasterPure™ Complete DNA & RNA Purification Kit (Biosearch Technologies, UK). Genomic DNA was sequenced in parallel on the MinION or Flongle using the Ligation Sequencing Kit, SQK‐LSK109 (Oxford Nanopore Technologies, UK) and NovaSeq 6000 or NextSeq 2000 using the Nextera XT library preparation kit (Illumina, USA).

### Bioinformatic analysis

To generate complete genomes, Unicycler v0.5.1 [27] in hybrid assembly mode, combining short reads and long reads, was used. Prior to assembly, the paired-end short reads were trimmed using fastp v0.24.0 [28], and long-read sequences were filtered using Filtlong v0.2.1 (https://github.com/rrwick/Filtlong) to ensure high-quality sequences (retaining sequences > 1 kbp). Whole genomes were annotated using RAST with default settings (https://rast.nmpdr.org/).

The multi-locus sequence type (MLST) and *emm* type were determined using the PubMLST database (https://pubmlst.org) [29].

Genetic relatedness among isolates was determined using whole genome MLST (wgMLST) with Bionumerics v8.1 (1998 loci, bioMérieux, France) and visualised in a minimum spanning tree. A maximum likelihood phylogenetic tree was constructed based on the core genome alignment generated with Prokka v1.14.6 [30], Roary v3.12.0 [31], and RAxML-NG [32] using the GTR+G model and 500 bootstrap support. The resulting tree was visualised and refined with iTOL v6.9.1 [33] and Inkscape v1.3.2. For SNP analysis, trimmed short paired-end reads from rGAS isolates were mapped against the complete genomes of the initial iGAS isolates using the Geneious mapper (Geneious Prime 2024.0.7, Dotmatics, USA) and snippy v4.6.0 [34]. Variant calling was performed with Freebayes v1.1.0, retaining only SNPs supported by a minimum of 10 reads with a mapping quality > 20.

To investigate differences in gene content, whole genome alignments were performed using circularised genome assemblies with MAUVE v1.1.3 [35] and visualised with Easyfig v2.25 [36] at default BLASTn parameters.

Virulence-related determinants were predicted using ABRicate v1.0.1 (https://github.com/tseemann/abricate) in conjunction with the Virulence Factor Database (VFDB) [37]. Additionally, the distantly related *sic* gene (*drs*12.01 allele) was included in our analysis as the *sic* gene present in the VFDB is specific to *emm*1, whereas the *drs* gene is associated with *emm*12. A threshold of 70% coverage was applied. Prior to the search, draft genomes were assembled *de novo* using SPAdes v4.0.0, and assemblies with a sequence length exceeding 2 Mbp were excluded.

Integrative and conjugative elements (ICEs) and prophage regions were identified with the ICEfinder detection tool from the ICEberg v3.0 database [38] and PhiSpy v4.2.21 tool [39], respectively. The amino acid sequences that are part of the ICE elements were compared to the UniProt database. The presence of predicted ICE regions with T4SS genes in other bacterial species was then searched in the NCBI database.

The prevalence of identified virulence determinants and T4SS components was compared using ABRicate v1.0.1 against a global collection of noninvasive and invasive *S. pyogenes* genomes corresponding to the *emm* types in this study: *emm*1 (*n* = 2073), *emm*12 (*n* = 1368), and *emm*89.0 (*n* = 62), sourced from previously published studies (Supplementary Table S14), further referred to as the comparative genomic dataset.

Additionally, 27 M1_UK_ lineage-defining single-nucleotide polymorphisms (SNPs) [11] were identified by mapping short reads to the *S. pyogenes* reference genome MGAS5005 (GenBank CP000017).

Lastly, to identify mutations linked to increased virulence in iGAS isolates from patients C and D, a comparative analysis with the non-invasive *emm*12 strain MGAS9429 (GenBank CP000259) was performed using Snippy v4.6.0. The prevalence of detected mutations in the comparative genomic dataset was analysed with BLASTn as described by Bah *et al.* [40]. Briefly, the start and end coordinates of the top hits were converted into BED files using BEDTools v2.31.1 [41] to retrieve corresponding nucleotide sequences from the genome assemblies. Subsequently, these sequences were translated into amino acid sequences, and variants were identified by comparison with the amino acid sequences of the reference proteins.

## Results

During the study period, a total of 31 cases of iGAS were identified, with four cases (13.0%) complicated by rGAS.

### Patient demographic

Two females (Patients A and D) and two males (Patients B and C) experienced recurrent GAS infections after initial iGAS. The median age of these patients was 67.5 years (range: 46–77 years), and their median Charlson comorbidity index was 4.5 (range: 1–8, Supplementary Table S3).

### Clinical presentation and microbiology findings

In all cases, the initial presentation of iGAS was a skin and soft tissue infection. All patients required ICU admission during their primary hospital stay. Except for Patient B, all patients required re-admission for rGAS. The median interval between the primary infection and the recurrence was 25.5 days (range 9–98 days).

The GAS strains from Patient A were resistant to erythromycin and clindamycin. The remaining GAS strains were sensitive to all tested antibiotics (Supplementary Table S4).

#### Patient A

The initial presentation of iGAS in patient A (female, age 69) involved upper limb necrotising fasciitis with a preceding chronic skin fissure on the finger. The recurrence manifested as cellulitis affecting the same upper limb region. The interval between the primary infection and recurrence was 19 days. No additional hospitalisations were reported between the primary and recurrent infections.

GAS was cultured from pus, wound swab, and tissue samples during the initial episode, whereas blood cultures were negative. Initial wound cultures also revealed the co-presence of *Staphylococcus aureus*. During the recurrent episode, blood cultures were not performed, and GAS was cultured from a wound swab.

#### Patient B

Patient B (male, age 46) initially presented with upper limb necrotising fasciitis complicated by streptococcal toxic shock syndrome without reported wound or injury. The first recurrence (after an interval of 9 days) also involved cellulitis in the same upper limb region.

Patient B showed GAS culture positivity in pus, wound swab, and blood cultures during the iGAS episode. However, during the rGAS episode, GAS was cultured only from a wound swab, as blood cultures were not conducted.

Notably, a second recurrence of cellulitis was reported; however, no microbiological cultures were performed. Based on the clinical response to linezolid treatment during the second recurrence, GAS aetiology was presumed, though the involvement of another gram-positive pathogen could not be excluded.

#### Patient C

Patient C (male, age 66) initially presented with foot gangrene accompanied by bloodstream infection.. The recurrence manifested as a foot surgical site infection with bloodstream involvement, occurring 98 days after the initial infection.

The patient had positive GAS cultures from both blood and wound swab samples in both episodes. The initial blood culture was also positive for *S. aureus*, while a wound swab taken during the recurrent episode revealed a co-infection with *Pseudomonas aeruginosa*.

Between the initial iGAS and recurrence, the patient was hospitalised twice for infectious skin and soft tissue complications involving the same foot region. These infections were caused by non-GAS pathogens, including *Staphylococcus haemolyticus*, *S. aureus*, *Enterococcus faecalis*, *Enterococcus raffinosus*, *Corynebacterium striatum*, *Escherichia coli*, *P. aeruginosa*, and *Anaerococcus murdochii.*

#### Patient D

Patient D (female, age 69) initially presented with an upper limb skin abscess and cellulitis, complicated by bloodstream infection. She reported a fall prior to infection. The recurrence, which occurred after 32 days, presented as upper limb cellulitis with concurrent septic gonitis. The patient exhibited GAS positivity in purulent material from an abscess during both episodes and in joint fluid during the rGAS episode. Blood cultures were positive during the initial episode, but were not performed during the recurrent episode. Patient D had a superinfection with ESBL-producing *E. coli* in the wound during the first episode and a soft tissue co-infection with the same species in the recurrent episode.

Between the initial iGAS and recurrence, the patient was hospitalised once for an upper limb wound infection caused by ESBL-producing *E. coli*.

**Figure 1.**
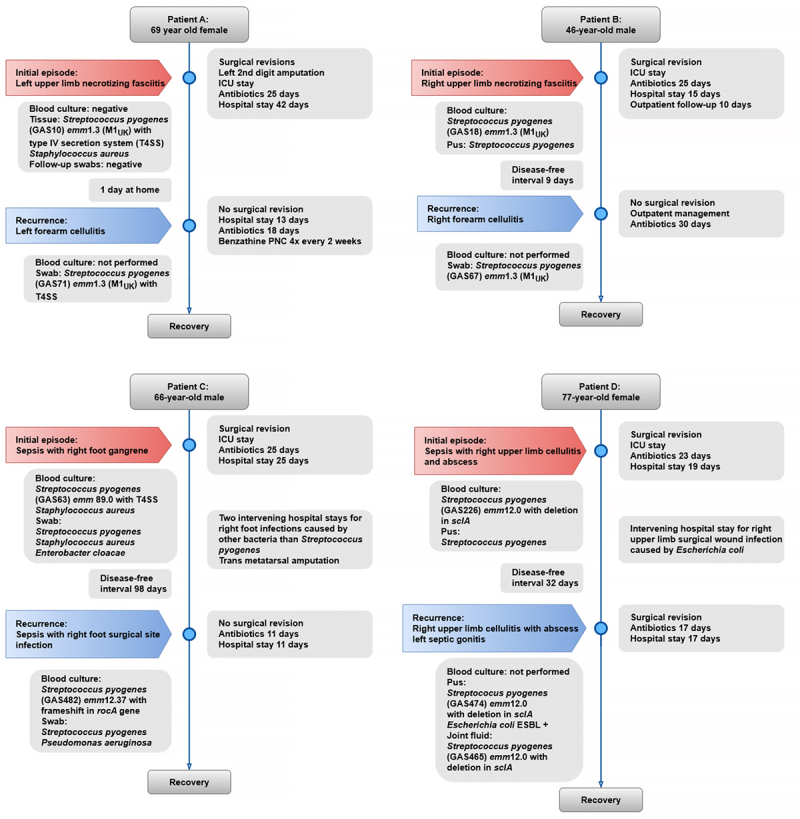
A–D: Timeline of management of patients with recurrent GAS infection.

A summary of the demographic and clinical data is presented in Table 1. Timelines for each case are illustrated in Figure 1A-D. A detailed description of the individual cases is provided in Supplementary Case Reports.

**Table 1.** Summary of demographic and clinical data in patients with GAS recurrences.

Characteristics	Patient A	Patient B	Patient C	Patient D
*Demographic data*				
Age (years)	69	46	66	77
Sex	Female	Male	Male	Female
Charlson comorbidity index	2	1	7	8
*Initial iGAS*				
GAS *emm* type	*emm*1.3	*emm*1.3	*emm*89.0	*emm*12.0
Type of iGAS	Necrotizing fasciitis	Necrotizing fasciitis, toxic shock syndrome	Sepsis	Sepsis
ICU stay	Yes	Yes	Yes	Yes
Surgery	Yes	Yes	Yes	Yes
Combination of antibiotics	Yes	Yes	Yes	Yes
Duration of hospital stay (days)	42	15	25	19
Duration of antibiotic therapy (days)	25	25	25	23
*Recurrence of GAS infection*				
GAS *emm* type	*emm*1.3	*emm*1.3	*emm*12.37	*emm*12.0
Number of recurrences	1	1	1	1
Hospitalisation	Yes	No	Yes	Yes
Type of infection	Cellulitis	Cellulitis	Sepsis, wound infection	Cellulitis, gonitis
Time to recurrence (days)	19	9	98	32
Duration of antibiotic therapy (days)	18	30	11	17

### Other laboratory findings

The median absolute leukocyte count during initial iGAS episodes was 20.5 × 10^9/L (range: 13.3–39.1 ×10^9/L), and the median CRP level was 286.5 mg/L (range: 224–390 mg/L) in the initial iGAS episodes. In contrast, during recurrent GAS episodes, these values were lower: the median leukocyte count was 15 × 10^9/L (range: 12.9–23.4 ×10^9/L), and the median CRP level was 76.2 mg/L (range: 50–268 mg/L). More detailed information is available in Supplementary Table S5. Immunology testing was performed at the following intervals after the initial presentation: 14 months (Patient A), 14 months (Patient B), 12 months (Patient C) and 10 months (Patient D). Immunology testing showed no abnormalities in Patients A and D, while deficiencies in mannose-binding lectin (MBL) and CD19+ lymphocytes were identified in Patients B and C (Table 2). Genetic testing did not detect any pathogenic variants indicative of primary immunodeficiency. However, the *MBL2* gene, which encodes the mannose-binding lectin, was not included in the genetic panel (Supplementary Table S1).

**Table 2.** Immunological results in patients A-D, pathological results are in bold, timing of testing: patients A and B – month 14, patients C – month 12, Patient D – month 10 after initial iGAS.

	Patient A	Patient B	Patient C	Patient D	Normal values
*Lymphocytes*					
CD3+ relative	85.0%	87.0%	81.0%	72.0%	50.0–91.0%
CD3+ absolute	3.73x10^9^/L	2.01x10^9^/L	0.91x10^9^/L	1.15x10^9^/L	0.78–3.00x10^9^/L
CD3-CD16,56+ relative	**4.0%**	8.0%	8.0%	12.0%	6.0–33.0%
CD3-CD16,56+ absolute	0.18x10^9^/L	0.18x10^9^/L	0.09x10^9^/L	0.19x10^9^/L	0.05–1.00x10^9^/L
CD4+ relative	42.0%	38.0%	47.0%	55.0%	28.0–64.0%
CD4+ absolute	1.84x10^9^/L	0.88x10^9^/L	0.53x10^9^/L	0.88x10^9^/L	0.50–2.00x10^9^/L
CD8+ relative	39.0%	**43.0%**	27.0%	13.0%	12.0–40.0%
CD8+ absolute	**1.71x10**^**9**^**/L**	0.99x10^9^/L	0.30x10^9^/L	0.21x10^9^/L	0.20–1.20x10^9^/L
CD19+ relative	9.0%	**3.0%**	**3.0%**	10.0%	7.2–22.5%
CD19+ absolute	0.40x10^9^/L	**0.07x10**^**9**^**/L**	**0.03x10**^**9**^**/L**	0.16x10^9^/L	0.10–0.60x10^9^/L
CD4+/CD8+ ratio	1.1	**0.9**	1.7	**4.2**	1.0–3.0
*Immunoglobulins*					
IgG	**18.50 g/L**	12.30 g/L	12.80 g/L	**14.30 g/L**	7.65–13.60 g/L
IgA	1.46 g/L	2.69 g/L	**6.39 g/L**	**3.99 g/L**	0.91–2.90 g/L
IgM	1.47 g/L	0.76 g/L	1.36 g/L	1.30 g/L	0.47–1.95 g/L
*Complement*					
C3	1.42 g/L	0.99 g/L	1.29 g/L	1.07 g/L	0.83–2.25 g/L
C4	**0.43 g/L**	0.23 g/L	0.30 g/L	0.23 g/L	0.14–0.35 g/L
Classical pathway*	104%	104%	109%	113%	69–129%
MBL pathway*	86.2%	**0.0%**	**1.7%**	44.6%	> 10.0%
Alternative pathway*	103.6%	84.3%	110.0%	**115.6%**	30.0–113.0%

*Classical, MBL and alternative pathway: percentage of activity (ELISA).

### Treatment

The median duration of antibiotic treatment for the initial iGAS infection was 25 days (range: 23–25 days), while for the recurrent infection, it was 17.5 days (range: 11–30 days). All patients were treated with beta-lactam during their initial episode. Moreover, in three out of four patients, linezolid was added. Thus, all patients received antibiotic treatment that was presumed to be curative. Patients A and D were treated with clindamycin prior to admission for their initial infection. Patient A received a secondary prophylactic course of benzathine penicillin G following the GAS recurrence. A detailed overview of antimicrobial treatment for individual patients is provided in Supplementary Table S6. Only Patient A received treatment with intravenous immunoglobulins during the first hospitalisation.

### Outcome

All patients recovered; no deaths were reported. Patients A and B were evaluated in the clinic 14 months after the initial episode. Both were clinically stable with completely healed wounds. At the follow-up visit at month 12, patient C was clinically stable with a granulating, non-inflamed chronic foot wound. Patient D was seen 10 months after the initial episode and was stable with the healed wound.

### *Characterisation of causative* S. pyogenes *isolates*

A total of nine *S. pyogenes* isolates were characterized.

For Patient A, two isolates were sequenced: GAS10 (deep wound swab from the initial episode) and GAS71 (wound swab from the recurrence). Both isolates were *emm*1 and ST28 and were identical using wgMLST (zero allele differences). In the isolate from the recurrence (GAS71), SNP analysis identified a substitution in the mobile element (IS*1548* family transposase), located 177 bp upstream of the laminin-binding surface protein. Additionally, a single adenine residue was deleted in the intergenic region, 121 bp upstream of the *speA* exotoxin gene.

Patient B also had two isolates: GAS18 (blood culture from the initial episode) and GAS67 (wound swab from the recurrence). Both isolates were *emm*1 and ST28 and were identical using wgMLST (zero allele differences) and SNP analysis (zero SNPs in the coding sequences).

**Figure 2.**
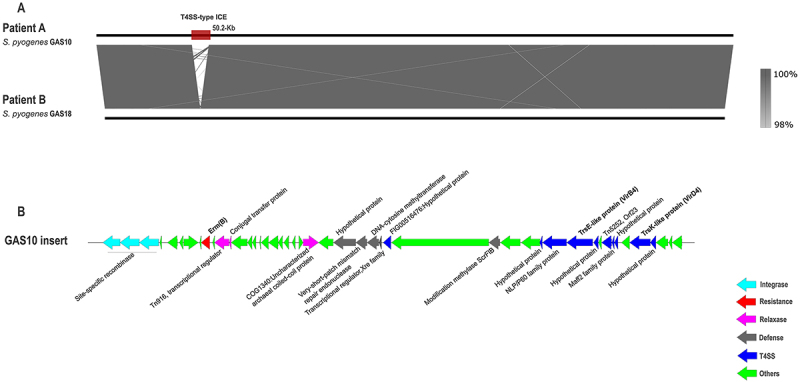
A: Genome comparison of *Streptococcus pyogenes* emm1.3 isolates from patients A and B. The grey regions between the genome maps indicate the similarity range of the nucleotide sequence. B: schematic representation of the identified insert (50.2 kb) in GAS10 isolate from Patient a carrying a T4SS-type integrative conjugative element with the *ermB* gene. The functional categories of genes are colour-coded, as shown in the legend.

All four isolates from Patients A and B contained the 27 M1_UK_ lineage-defining SNPs and carried a similar spectrum of virulence genes, which were present in 97–100% of the comparative genomic dataset (Figure 2A). In contrast, the *sic* gene was identical (933 bp) in all four *emm*1 isolates from this study, whereas high variability in the gene sequence was observed in the comparative genomic dataset (Supplementary Table S2).

In the isolates from Patient A, the ICE with a triple serine integrase module (site-specific recombinases) was located within the Type I restriction-modification system (*hsdM* genes). Additionally, the insert contained the methylase gene *ermB*, integrated *via* Tn*916*-related elements, along with genes encoding T4SS (Figure 2B, GenBank PV074749). The predicted ICE region demonstrated 99.9% sequence similarity and coverage (Mash distance 0.0016) with the ICE_SsalL22_*hsdM* element (50 kb) from *Streptococcus salivarius* [42], differing only in the lengths of the Tn*916* transcriptional regulator and a hypothetical protein. Among the 250 top BLASTn hits from the NCBI database, only four were *S. pyogenes* strains (USA, GenBank CP118307.1, CP118308.1, CP118309.1, CP118481.1) carrying the complete ICE element with 100% coverage and 99% identity; the remaining sequences corresponded to other *Streptococcus* species from *S. agalactiae* (China, GenBank CP138371.1), and *S. pneumoniae* (China, GenBank CP137105.1). No exact match with complete ICE elements was found in the comparative genomic dataset. Detailed annotation of the T4SS-type integrative conjugative element in the GAS10 strain of Patient A is available in Supplementary Table S7.

For Patient C, the paired isolates GAS63 and GAS482, both derived from the blood cultures (iGAS and recurrent iGAS infections), were genetically distinct. GAS63 belonged to *emm*89 and ST101, while GAS482 was classified as *emm*12.37 and ST36. The wgMLST analysis revealed 1,368 allelic differences between the two isolates (Supplementary Figure S1).

In the initial episode, isolate (GAS63) from Patient C, a unique ICE element (46,132 bp, GenBank PV074750, now named ICESpy63) was identified, which did not show an exact match in the ICEberg v3.0 database. This mobile element contained genes associated with the T4SS, including VirB4 and VirD4. Additionally, the ICE harboured an uncharacterized major facilitator superfamily (MFS) transporter, which might confer resistance to antimicrobial agents of unknown specificity. The element also carried genes encoding collagen-binding adhesin proteins (Cna B-type domain-containing protein), which are essential for host interaction. Top BLASTn hits from the NCBI database revealed 94% similarity and 93% coverage with *S. pyogenes* (USA, GenBank CP060638.1) and a 93% similarity and 82% coverage with *S. anginosus* (USA, GenBank CP068060.1). No exact match with complete ICE elements was found in the comparative genomic dataset. Detailed annotation of the T4SS-type integrative conjugative element in the GAS63 strain of Patient C is available in Supplementary Table S8.

**Figure 3.**
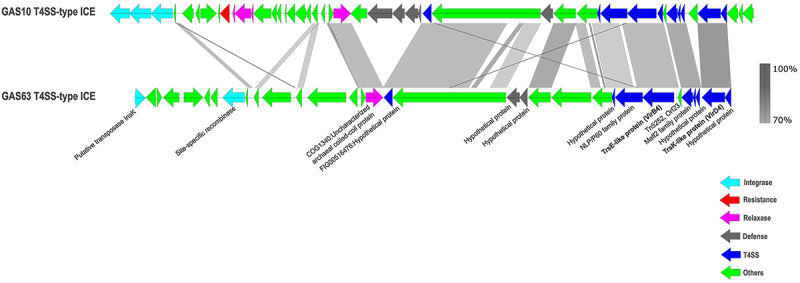
Pairwise linear comparison of the predicted T4SS-type ICEs found in GAS10 (*emm*1) and GAS63 (*emm*89.0) isolates. The grey regions indicate nucleotide sequence similarities ranging between 70% and 100%.

Due to the identification of T4SS in two isolates (GAS10 Patient A and GAS63 Patient C), the ICE regions were compared by pairwise sequence alignment and variations in the integrase and accessory genes were observed (Figure 3). In contrast, the core T4SS-related genes, ATPase VirB4 and Type IV Coupling Protein VirD4, showed sequence similarities of 78% and 85%, respectively (Figure 3). Screening of T4SS genes across representative global isolates indicated a near absence to a low-frequency occurrence (0.14% − 13%) in isolates from Europe, Australia and the USA (Supplementary Table S9). Conversely, these genes were detected in 64–85% of Chinese isolates and 49–73% of Hong Kong isolates, with sequence similarity of 80% and 85% for the VirB4 and VirD4 genes, respectively.

The spectrum of virulence genes analyzed using VFDB differed between the GAS63 and GAS482 isolates from Patient C due to their distinct *emm* types (*emm*89.0 and *emm*12.37, respectively). However, the distribution of these genes was consistent with the frequencies observed for their respective *emm* types in the comparative genomic dataset. The only exception was GAS482, which lacked the *drs* gene (Supplementary Figure S2).

Patient D had three isolates: GAS226 (blood culture from the initial iGAS infection), GAS465 (joint fluid from the first rGAS infection), and GAS474 (wound swab from the second rGAS infection). All three isolates belonged to *emm*12 and ST36. The isolates were identical by wgMLST (zero allele differences) and SNP analysis (zero SNPs in the coding sequences). A similar spectrum of virulence genes was observed compared to the global genome data set, with high gene variability observed in the *drs* gene (Supplementary Table S2).

To identify putative genetic determinants of virulence in *emm*12 isolates, we compared isolates from Patient C and Patient D with the non-iGAS *emm*12 reference genome MGAS9429. SNP analysis revealed over 60 non-synonymous mutations (Supplementary Tables S10 and S11). Considering putative virulence factors, both iGAS isolates showed mutations in genes for oxidative stress response (*msrA*, and *sodA*), dnase streptodornase (*sda*1), *sagG* family ABC transporter permease subunit, and penicillin-binding protein (PBP2X). However, these mutations were also found in high prevalence in the comparative genomic dataset (Supplementary Table S12).

Further, both strains showed sequence variability in genes encoding collagen-like surface proteins (*sclA* and *sclB*). In the reference strain MGAS9429, the *sclA* gene encodes a protein containing seven conserved “PGEKAPEKS” repeat sequences within the linker region connecting the collagen-like domain to the cell wall membrane domain. Three *emm*12 isolates from patient D harboured a 79–amino acid deletion within this linker region, also reducing the number of “PGEKAPEKS” repeats to only three. This deletion was rarely present in *emm*12 isolates from the USA (1.4%, 5/354), although partial fragments were common. The presence of the 79-aa deletion was undetermined for certain isolates (UK; 18/516 and Portugal; 1/12), as the SPAdes assembly from Illumina short-read data yielded only fragmented *sclA* sequences, most likely due to complex internal repeat regions that impeded accurate assembly. The *emm*1 isolates showed longer deletions due to natural variation in repeat number.

The *sclB* gene displayed a frameshift mutation resulting from the duplication of “AACAA” repeats located directly downstream of the start codon (GTG) in *emm*12 strains from both Patient C and Patient D. As reported by Bao *et al*., premature translation termination of the *sclB* gene is unlikely linked to increased virulence [43], therefore, comparative analysis with the genomic dataset was not performed.

Additionally, the GAS482 isolate from Patient C carried a frameshift mutation (p.N419fs) in the *rocA* gene, a transcriptional regulator of CovRS and a putative regulator of a broader gene network [44], and a missense mutation (p.V29A) in the carboxypeptidase penicillin-binding protein (PBP3). The RocA frameshift variant in GAS482 was found only in six isolates in the comparative genomic data set; four were noninvasive *emm*12 isolates from the UK, and two were non-invasive *emm*12 isolates from the Czech Republic (Supplementary Table S12). The PBP3 variant identified in our study was found in 21.8% *emm*12 isolates (52/239) collected from Denmark, 2.5% (13/516) from the UK, and 17.94% (7/39) from the Czech Republic (Supplementary Table S12).

**Figure 4.**
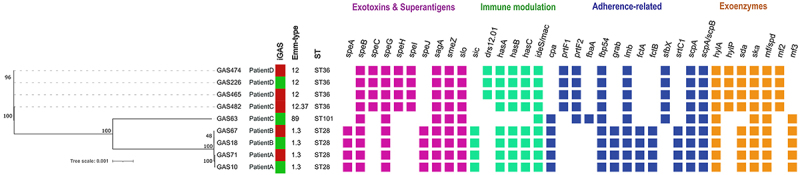
Core-genome maximum-likelihood phylogenetic tree inferred with GTR+G model with 500 bootstrap replicates. Green and red squares indicate the initial and recurrent episode isolates. The presence of virulence genes predicted with the VFDB database is classified into four categories and is shown as colored squares.

The schematic representation of virulence genes in all isolates is shown in Figure 4. The minimum spanning tree constructed from the wgMLST analysis results of all isolates in the study is shown in Supplementary Figure S1.

Antimicrobial resistance was observed only in the isolates from Patient A, with resistance to erythromycin and clindamycin, mediated by the *ermB* methylase gene integrated by the Tn*916*-related elements.

## Discussion

This case series presents four patients with severe iGAS complicated by subsequent rGAS infections despite seemingly adequate surgical debridement and antibiotic treatment during the initial episode.

The recurrences occurred in 12.9% of our patients with iGAS. However, the overall rate of recurrent infections following iGAS remains unclear and may not differ from recurrence rates complicating non-invasive infections. Limited data addressing this topic is available. For instance, during a prolonged outbreak of GAS infections in a U.S. nursing home, Dooling *et al*. documented 15 invasive infections, including three recurrences (20%) [45]. GAS recurrent infections typically include erysipelas/cellulitis, tonsillopharyngitis and vulvovaginitis. In our cohort, three patients had cellulitis (in one patient complicated with gonitis), and one patient had sepsis.

Recurrent GAS bacteremia was identified only in Patient C in our cohort, as blood cultures were not performed during the rGAS episodes in patients A, B and D. Several case reports describe recurrent episodes of GAS bacteremia. Rasmussen reported a case of recurrent GAS bacteremia with the same *emm* type (*emm*87) in an elderly man with multiple medical comorbidities who failed to develop a specific opsonising antibody response [23]. Hattori *et al*. described a case of an immunocompromised child with T-cell and B-cell dysfunction who had recurrent GAS bacteremia caused by different *emm* types (*emm*1 and *emm*12) [22]. Similarly, Gazzaz *et al*. reported recurrent bacteremia in a child with no prior medical history or immune dysfunction, involving GAS *emm*4 and *emm*1 strains [46]. In our cohort, the two episodes of bacteremia in Patient C were caused by different *emm* types (*emm*89 and *emm*12), suggesting reinfection due to increased host susceptibility. Patient C had multiple medical comorbidities similar to the case described by Rasmussen [23] and, in line with the pediatric case above, was found to be immunocompromised, exhibiting MBL pathway and CD19+ lymphocyte deficiencies. Additionally, the presence of a chronic wound likely served as a persistent portal of entry, facilitating reinfection with a different *emm* type. On the other hand, both *S. pyogenes* strains carried distinct features associated with presumptive increased virulence, including the presence of T4SS in *emm*89.0 and a frameshift mutation in the *rocA* gene in *emm*12.37.

Of the 93 patients with lower limb necrotising fasciitis (with bacterial aetiology not specified) reported in Traineau *et al*., 32 (35.1%) had recurrences, of which 28 (87.5%) were non-necrotising (non-invasive) and 4 (12.5%) were necrotising (invasive) [47]. Indeed, cases of recurrent necrotising fasciitis caused by GAS are sporadic. Kuzdan *et al*. described a child with hereditary sensory and autonomic neuropathy type IV who experienced three episodes of recurrent necrotising fasciitis, with the last one proving fatal. The strains from the final two episodes were identical according to arbitrarily primed PCR (AP-PCR), but the *emm* types were not specified [48]. Our two patients (A and B) with upper limb necrotising fasciitis had recurrences caused by the same *emm* type (*emm*1), suggesting relapses. However, unlike Kuzdan *et al*., and in line with Traineau *et al*. [47,48], the recurrent episodes were non-invasive (superficial cellulitis), and both patients survived.

In three out of four of our patients, upper limb involvement was present. This is quite uncommon, as lower limb cellulitis is more usual. As described in the Supplementary case reports, Patient A had a chronic skin fissure on the finger; Patient B did not report any identifiable portal of entry; and Patient D experienced a fall prior to infection, resulting in a subcutaneous hematoma in the upper limb, which may have served as a predisposing factor. None of the patients had a recent vaccination or injection at the affected site.

Despite the documented high mortality rates associated with severe iGAS [49,50], none of the patients in our series died. This favourable outcome may be attributed to heightened awareness during the ongoing GAS epidemic in the Czech Republic, rapid identification of GAS, combination antibiotic therapy, prompt ICU admission, and timely surgical debridement. Nevertheless, these comprehensive measures failed to prevent recurrences in our patients.

Apart from surgical debridement, all patients were treated with antibiotics for at least 21 days during their initial episode. High-dose penicillin G (or another beta-lactam) was administered as a backbone antibiotic for at least part of their clinical course, as all strains were exquisitely sensitive to it (MIC ≤0.06 mg/L).

To suppress toxin and virulence factor production by inhibiting protein synthesis, linezolid was administered to three out of four patients during the initial episode of infection. It was chosen empirically over the more commonly recommended clindamycin due to concern about possible clindamycin resistance, later confirmed only in the isolate from Patient A. None of the bacterial strains were resistant to linezolid. The impact of possible “de-escalation” to clindamycin on patient outcomes remains uncertain.

In Patient A, clindamycin resistance was clinically significant, as the patient received clindamycin empirically for one day prior to hospital admission. This ineffective treatment likely contributed to a poorer outcome. Conversely, Patient D received clindamycin prior to admission, and the strain was sensitive. However, this treatment did not prevent the need for hospital admission or the development of iGAS.

Multiple factors influence the response of GAS infection to antibiotics, including beta-lactamases production by resident or co-infecting bacteria, biofilm formation, intracellular persistence of bacteria, and impaired delivery of antibiotics and immune cells due to tissue edema, necrosis and vascular thrombosis [51]. Bacterial co-infections or superinfections were present in three of four patients, potentially contributing to recurrences.

Secondary antibiotic prophylaxis has been shown to prevent recurrent leg cellulitis [19,52]. In our study, one patient (Patient A) received secondary prophylaxis with benzathine penicillin G following rGAS. However, whether secondary antibiotic prophylaxis should be recommended for all patients with iGAS remains unresolved and warrants future investigation.

Host factors can play a role in increasing the risk of recurrent infection. In our study, two patients (B and C) were found to have lectin-pathway (mannose-binding lectin, MBL) deficiency upon subsequent immunology testing. MBL pathway deficiency, which affects 5–7 % of the Caucasian population, has been associated with an increased risk of severe bacterial infections [53,54]. Our findings suggest that recurrent GAS infection in adults could indicate an undiagnosed MBL pathway complement deficiency.

Additionally, both patients also exhibited low levels of CD19+ B lymphocytes despite having normal immunoglobulin levels. While the clinical significance of this finding remains unclear, both primary and secondary B-cell deficiencies are established risk factors for infections [55–57].

The concurrent presence of low lectin pathway activity and CD19^+^ lymphocyte deficiency in Patients B and C is noteworthy. Although no direct mechanistic link between these two immunological abnormalities has been established, their co-occurrence may synergistically increase susceptibility to bacterial infections. In contrast, the other two patients (A and D) had no discernible immune abnormalities. While this observation could be coincidental due to the limited sample size, it highlights a potential area for further investigation.

*S. pyogenes* is known for producing various virulence factors contributing to a broad spectrum of infections, and certain *emm* types were suggested to have increased virulence, e.g. *emm*1 M1_UK_ lineage [7,58]. However, GAS strains causing iGAS and rGAS in our patients belonged to three different *emm* types (1, 12 and 89). Comparative analysis of virulence genes identified in VFDB and the global collection of isolates (including non-invasive strains) found the presence of virulence genes to be *emm*-specific, with no association to infection type (iGAS or rGAS). Genomic comparisons of the same *emm*-types further supported these findings.

In contrast to the consistent presence of virulence genes included in the VFDB, unique molecular determinants or mutations suggesting increased virulence or predisposition to recurrence were identified. In Patients A and C, the presence of ICE carrying the T4SS genes was identified, and was relatively rare except in *emm*12 isolates from China and Hong Kong, but with 80% and 85% sequence similarity in the VirB4 and VirD4 genes of the integrative conjugative element. All four *S. pyogenes* strains identified through BLAST search in the NCBI database carried the ICE element nearly identical to that found in the GAS10 *emm*1 strain in our study. These included three invasive *emm*82/ST36 isolates, which had displayed recombinational switching of the *emm* region genes, and one invasive *emm*12/ST36 strain, all originating from the same study [59]. Secretion systems, such as T4SS, once thought to be exclusive to Gram-negative bacteria, have also been identified in Gram-positive species, including *Streptococcus suis* [60]. T4SS enables the injection of bacterial proteins into host cells, playing a critical role in pathogenesis. Therefore, the presence of T4SS in isolates from two of our patients may have contributed to the development of rGAS, and the role of T4SS variants on *S. pyogenes* virulence should be further investigated.

We also observed the genetic changes between the initial episode and recurrence isolates from Patient A (GAS10 and GAS71). However, it is unclear whether mutations upstream of the laminin-binding surface protein and *speA* exotoxin, identified in the isolate from the recurrence (GAS71), may impact pathogenicity due to their distant locations, 177 bp and 121 bp upstream of the genes, respectively, and further investigation is needed. Notably, Patient A’s rGAS episode was milder than the initial iGAS episode, with no need for surgical intervention.

Finally, comparative analysis of the *emm*12 isolates (Patients C and D) and the non-invasive comparative strain revealed several non-synonymous SNPs in genes associated with virulence. The 79–amino acid deletion observed in the SclA protein sequence of the isolates from Patient D was largely absent in the comparative dataset. While the precise functional significance of this deletion remains unclear, structural changes in the *sclA* gene have been linked to virulence. For example, variations in the SclA protein, such as “PGEKAPEKS” repeats in the linker region, may influence strain binding to host molecules [61]. Additionally, truncations in the *sclA* gene have been linked to altered virulence in animal models [62,63].

A frameshift mutation in the *rocA* gene in Patient C’s *emm*12.37 strain likely upregulated virulence factor expression through the CovRS system and a putative larger gene network [44]. In serotype M18 Group A *Streptococcus*, a naturally occurring truncation of RocA has been shown to enhance capsule production, prolonged colonisation, and increased transmissibility [64]. However, the predicted length of the truncated protein differs between the M18 GAS strains and the M12 isolates from our study. These differences in protein structure may contribute to variations in the associated phenotypes and warrant further investigation.

Our study has several limitations. First, as a single-center retrospective case series without a control group (patients with non-recurrent iGAS, asymptomatic carriers or healthy volunteers), the generalizability of the findings is limited. Second, blood cultures were not performed in three out of four recurrent cases, thus precluding the potential identification of recurrent bacteremia. Although *S. pyogenes* isolates from initial episodes and recurrences in Patients A, B and D were identical by wgMLST, the reinfection could not be ruled out. Furthermore, only Patient A in our cohort received intravenous immunoglobins (IVIG). Given the reported effectiveness of IVIG in patients with streptococcal toxic shock syndrome [65], its use might have improved outcomes in our cohort. These limitations highlight the need for further research to enhance our understanding and management of iGAS and recurrent GAS infection.

## Conclusion

This study highlights four cases of severe iGAS complicated by recurrent infection despite extended antibiotic treatment and surgical debridement. Our findings suggest that the interplay between bacterial virulence factors (e.g. the presence of T4SS, sequence variability in genes encoding collagen-like surface protein (SclA) and mutations in transcriptional regulator RocA) and host immunological deficiencies (e.g. MBL deficiency, low CD19+ lymphocytes) may increase the risk for recurrence. Future research should focus on improving the management of iGAS to enhance survival and prevent recurrences in at-risk populations.

## Ethics

The study was approved by the Ethics Committee of University Hospital Motol and the Second Faculty of Medicine, Charles University, Prague, Czech Republic, with Approval Number EK-579/23. The study adheres to the Declaration of Helsinki.

## Supplementary Material

Supplementary_Tables_revised2_final.xlsx

Supplementary figures and tables legends.docx

## Data Availability

Raw sequence data generated in this study are deposited in the Sequence Read Archive under BioProject PRJNA1218789. The nucleotide sequences of ICE elements found in GAS10 and GAS63 strains were submitted to GenBank under accession numbers PV074749 and PV074750.

## References

[1] Alcolea-Medina A, Snell LB, Alder C, et al. The ongoing *Streptococcus pyogenes* (group A *Streptococcus*) outbreak in London, United Kingdom, in December 2022: a molecular epidemiology study. Clin Microbiol Infect. 2023;29(7):887–15. doi: 10.1016/j.cmi.2023.03.001 36925107 PMC10769882

[2] Gouveia C, Bajanca-Lavado MP, Mamede R, et al. Sustained increase of paediatric invasive *Streptococcus pyogenes* infections dominated by M1UK and diverse *emm*12 isolates, Portugal, September 2022 to May 2023. Euro SurveIll. 2023;28(36):2300427. doi: 10.2807/1560-7917.ES.2023.28.36.2300427 37676143 PMC10486195

[3] Guy R, Henderson KL, Coelho J, et al. Increase in invasive group A streptococcal infection notifications, England, 2022. Euro SurveIll. 2023;28(1):2200942. doi: 10.2807/1560-7917.ES.2023.28.1.2200942 36695450 PMC9817207

[4] Johannesen TB, Munkstrup C, Edslev SM, et al. Increase in invasive group A streptococcal infections and emergence of novel, rapidly expanding sub-lineage of the virulent *Streptococcus pyogenes* M1 clone, Denmark, 2023. Euro SurveIll. 2023;28(26):2300291. doi: 10.2807/1560-7917.es.2023.28.26.2300291 37382884 PMC10311951

[5] Rodriguez-Ruiz JP, Lin Q, Lammens C, et al. Increase in bloodstream infections caused by *emm*1 group A *Streptococcus* correlates with emergence of toxigenic M1UK, Belgium, May 2022 to August 2023. Euro SurveIll. 2023;28(36):2300422. doi: 10.2807/1560-7917.ES.2023.28.36.2300422 37676145 PMC10486196

[6] de Arellano ER, Saaverda-Lozano J, Villalón P, et al. Clinical, microbiological, and molecular characterization of pediatric invasive infections by *Streptococcus pyogenes* in Spain in a context of global outbreak. mSphere. 2024;9(3):e0072923. doi: 10.1128/msphere.00729-23 38440985 PMC10964401

[7] Beres SB, Olsen RJ, Long SW, et al. Increase in invasive *Streptococcus pyogenes* M1 infections with close evolutionary genetic relationship, Iceland and Scotland, 2022 to 2023. Euro SurveIll. 2024;29(13):2400129. doi: 10.2807/1560-7917.ES.2024.29.13.2400129 38551096 PMC10979525

[8] Wolters M, Berinson B, Degel-Brossman N, et al. Population of invasive group A streptococci isolates from a German tertiary care center is dominated by the hypertoxigenic virulent M1UK genotype. Infection. 2024;52(2):667–671. doi: 10.1007/s15010-023-02137-1 38064158 PMC10954911

[9] Lynskey NN, Jauneikaite E, Li HK, et al. Emergence of dominant toxigenic M1T1 *Streptococcus pyogenes* clone during increased scarlet fever activity in England: a population-based molecular epidemiological study. Lancet Infect Dis. 2019;19(11):1209–1218. doi: 10.1016/s1473-3099(19)30446-3 31519541 PMC6838661

[10] Davies MR, Keller N, Brouwer S, et al. Detection of *Streptococcus pyogenes* M1UK in Australia and characterization of the mutation driving enhanced expression of superantigen SpeA. Nat Commun. 2023;14(1):1051. doi: 10.1038/s41467-023-36717-4 36828918 PMC9951164

[11] Li HK, Zhi X, Vieira A, et al. Characterization of emergent toxigenic M1UK *Streptococcus pyogenes* and associated sublineages. Microb Genom. 2023;9(4):mgen000994. doi: 10.1099/mgen.0.000994 37093716 PMC10210942

[12] Li Y, Rivers J, Mathis S, et al. Genomic surveillance of *Streptococcus pyogenes* strains causing invasive disease, United States, 2016–2017. Front microbiol. 2020;11:1547. doi: 10.3389/fmicb.2020.01547 32849323 PMC7396493

[13] Bellés-Bellés A, Prim N, Mormeneo-Bayo S, et al. Changes in group A *Streptococcus emm* types associated with invasive infections in adults, Spain, 2023. Emerg Infect Dis. 2023;29(11):2390–2392. doi: 10.3201/eid2911.230857 37877666 PMC10617363

[14] Johnson CM, Grossman AD. Integrative and conjugative elements (ICEs): what they do and how they work. Annu Rev Genet. 2015;49(1):577–601. doi: 10.1146/annurev-genet-112414-055018 26473380 PMC5180612

[15] Miller KM, Lamagni T, Cherian T, et al. Standardization of epidemiological surveillance of invasive group A streptococcal infections. Open Forum Infect Dis. 2022;9(Suppl 1):S31–S40. doi: 10.1093/ofid/ofac281 36128405 PMC9474937

[16] Davies HD, McGeer A, Schwartz B, et al. Invasive group A streptococcal infections in Ontario, Canada. Ontario group A streptococcal study group. N Engl J Med. 1996;335(8):547–554. doi: 10.1056/nejm199608223350803 8684408

[17] Steere AC, Lamagni T, Curtis N, et al. Invasive group A streptococcal disease epidemiology, pathogenesis and management. Drugs. 2012;72(9):1213–1227. doi: 10.2165/11634180-000000000-00000 22686614 PMC7100837

[18] Andreoni F, Zürcher C, Tarnutzer A, et al. Clindamycin affects group A *Streptococcus* virulence factors and improves clinical outcome. J Infect Dis. 2017;215(2):269–277. doi: 10.1093/infdis/jiw229 27247345

[19] Thomas KS, Crook AM, Nunn AJ, et al. Penicillin to prevent recurrent leg cellulitis. N Engl J Med. 2013;368(18):1695–1703. doi: 10.1056/nejmoa1206300 23635049

[20] Verkaeren E, Epelboin L, Epelboin S, et al. Recurrent *Streptococcus pyogenes* genital infection in a woman: test and treat the partner! Int J Infect Dis. 2014;29:37–39. doi: 10.1016/j.ijid.2014.07.026 25449232

[21] Mazón A, Gil-Setas A, Sota de la Gándara LJ, et al. Transmission of *Streptococcus pyogenes* causing successive infections in a family. Clin Microbiol Infect. 2003;9(6):554–559. doi: 10.1046/j.1469-0691.2003.00567.x 12848734

[22] Hattori T, Minami M, Narita K. Recurrent bacteremia with different strains of *Streptococcus pyogenes* in an immunocompromised child. J Infect chemother. 2016;22(6):421–423. doi: 10.1016/j.jiac.2015.12.014 26846458

[23] Rasmussen M. Recurrent sepsis caused by *Streptococcus pyogenes*. J Clin microbiol. 2011;49(4):1671–1673. doi: 10.1128/JCM.02378-10 21346045 PMC3122791

[24] Shaikh N, El-Menyar A, Mudali IN, et al. Clinical presentations and outcomes of necrotizing fasciitis in males and females over a 13-year period. Ann Med Surg. 2015;4(4):355–360. doi: 10.1016/j.amsu.2015.09.005 PMC4602355 26568823

[25] Tkadlec J, Peckova M, Sramkova L, et al. The use of broad-range bacterial PCR in the diagnosis of infectious diseases: a prospective cohort study. Clin Microbiol Infect. 2019;25(6):747–752. doi: 10.1016/j.cmi.2018.10.001 30321604

[26] Breiman RF, Davis JP, Facklam RR, et al. Defining the group A streptococcal toxic shock syndrome: rationale and consensus definition. JAMA. 1993;269(3):390–391. doi: 10.1001/jama.1993.03500030088038 8418347

[27] Wick RR, Judd LM, Gorrie CL, et al. Unicycler: resolving bacterial genome assemblies from short and long sequencing reads. PLoS Comput Biol. 2017;13(6):e1005595. doi: 10.1371/journal.pcbi.1005595 28594827 PMC5481147

[28] Chen S. Ultrafast one-pass FASTQ data preprocessing, quality control, and deduplication using fastp. Imeta. 2023;2(2):e107. doi: 10.1002/imt2.107 38868435 PMC10989850

[29] Jolley KA, Bray JE, Maiden MCJ. Open-access bacterial population genomics: BIGSdb software, the PubMLST.org website and their applications. Wellcome Open Res. 2018;3:124. doi: 10.12688/wellcomeopenres.14826.1 30345391 PMC6192448

[30] Seemann T. Prokka: rapid prokaryotic genome annotation. Bioinformatics. 2014;30(14):2068–2069. doi: 10.1093/bioinformatics/btu153 24642063

[31] Page AJ, Cummins CA, Hunt M, et al. Roary: rapid large-scale prokaryote pan genome analysis. Bioinformatics. 2015;31(22):3691–3693. doi: 10.1093/bioinformatics/btv421 26198102 PMC4817141

[32] Stamatakis A. RaXml version 8: a tool for phylogenetic analysis and post-analysis of large phylogenies. Bioinformatics. 2014;30(9):1312–1313. doi: 10.1093/bioinformatics/btu033 24451623 PMC3998144

[33] Letunic I, Bork P. Interactive tree of life (iTOL) v6: recent updates to the phylogenetic tree display and annotation tool. Nucleic Acids Res. 2024;52(W1):W78–W82. doi: 10.1093/nar/gkae268 38613393 PMC11223838

[34] Seemann T. Snippy: rapid haploid variant calling and core SNP phylogeny, 2020. Available from: https://github.com/tseemann/snippy

[35] Darling AE, Mau B, Perna NT. Progressive Mauve: multiple genome alignment with gene gain, loss and rearrangement. PLOS ONE. 2010;5(6):e11147. doi: 10.1371/journal.pone.0011147 20593022 PMC2892488

[36] Sullivan MJ, Petty NK, Beatson SA. Easyfig: a genome comparison visualizer. Bioinformatics. 2011;27(7):1009–1010. doi: 10.1093/bioinformatics/btr039 21278367 PMC3065679

[37] Liu B, Zheng D, Zhou S, et al. VFDB 2022: a general classification scheme for bacterial virulence factors. Nucleic Acids Res. 2022;50(D1):D912–D917. doi: 10.1093/nar/gkab1107 34850947 PMC8728188

[38] Wang M, Liu G, Liu M, et al. Iceberg 3.0: functional categorization and analysis of the integrative and conjugative elements in bacteria. Nucleic Acids Res. 2024;52(D1):D732–D737. doi: 10.1093/nar/gkad935 37870467 PMC10767825

[39] Akhter S, Aziz RK, Edwards RA. PhiSpy: a novel algorithm for finding prophages in bacterial genomes that combines similarity and composition-based strategies. Nucleic Acids Res. 2012;40(16):e126. doi: 10.1093/nar/gks406 22584627 PMC3439882

[40] Bah SY, Keeley AJ, Armitage EP, et al. Genomic characterization of skin and soft tissue *Streptococcus pyogenes* isolates from a low-income and a high-income setting. mSphere. 2023;8(1):e0046922. doi: 10.1128/msphere.00469-22 36507654 PMC9942559

[41] Quinlan AR, Hall IM. Bedtools: a flexible suite of utilities for comparing genomic features. Bioinformatics. 2010;26(6):841–842. doi: 10.1093/bioinformatics/btq033 20110278 PMC2832824

[42] Lao J, Guédon G, Lacroix T, et al. Abundance, diversity and role of ICEs and IMEs in the adaptation of *Streptococcus salivarius* to the environment. Genes (Basel). 2020;11(9):999. doi: 10.3390/genes11090999 32858915 PMC7563491

[43] Bao YJ, Liang Z, Mayfield JA, et al. Genomic characterization of a pattern D *Streptococcus pyogenes emm5*3 isolate reveals a genetic rationale for invasive skin tropicity. J Bacteriol. 2016;198(12):1712–1724. doi: 10.1128/JB.01019-15 27044623 PMC4886759

[44] Biswas I, Scott JR. Identification of *rocA*, a positive regulator of *covR* expression in the group A *Streptococcus*. J Bacteriol. 2003;185(10):3081–3090. doi: 10.1128/JB.185.10.3081-3090.2003 12730168 PMC154078

[45] Dooling KL, Crist MB, Nguyen DB, et al. Investigation of a prolonged group A streptococcal outbreak among residents of a skilled nursing facility, Georgia, 2009–2012. Clin Infect Dis. 2013;57(11):1562–1567. doi: 10.1093/cid/cit558 24021484

[46] Gazzaz N, Mailman T, Foster JR. Recurrent invasive group A streptococcal infection with four-limb amputation in an immunocompetent child. BMJ Case Rep. 2018;2018:bcr2018225292. doi: 10.1136/bcr-2018-225292 PMC6069930 30061131

[47] Traineau H, Charpentier C, Lepeule R, et al. First-year recurrence rate of skin and soft tissue infections following an initial necrotizing soft tissue infection of the lower extremities: a retrospective cohort study of 93 patients. J Am Acad Dermatol. 2023;88(6):1360–1363. doi: 10.1016/j.jaad.2022.12.044 36702443

[48] Kuzdan C, Soysal A, Altinkanat G, et al. Recurrent fatal necrotizing fasciitis due to *Streptococcus pyogenes* in a child with hereditary sensory and automic neuropathy type IV. Jpn J Infect Dis. 2011;64(2):147–149. doi: 10.7883/yoken.64.147. 21519130

[49] Björck V, Påhlman LI, Bodelsson M, et al. Morbidity and mortality in critically ill patients with invasive group A *streptococcus* infection: an observational study. Crit Care. 2020;24(1):302. doi: 10.1186/s13054-020-03008-z 32505194 PMC7275847

[50] Orieux A, Prevel R, Dumery M, et al. Invasive group A streptococcal infections requiring admission to ICU: a nationwide, multicenter, retrospective study (ISTRE study). Crit Care. 2024;28(1):4. doi: 10.1186/s13054-023-04774-2 38167516 PMC10759709

[51] Johnson AF, LaRock CN. Antibiotic treatment, mechanisms for failure, and adjunctive therapies for infections by group A *Streptococcus*. Front microbiol. 2021;12:760255. doi: 10.3389/fmicb.2021.760255 34803985 PMC8601407

[52] Dalal A, Eskin-Schwartz M, Mimouni D, et al. Interventions for the prevention of recurrent erysipelas and cellulitis. Cochrane Database Syst Rev. 2017;2017(6):CD009758. doi: 10.1002/14651858.cd009758.pub2 PMC6481501 28631307

[53] Brodszki N, Frazer-Abel A, Grumach AS, et al. European society for immunodeficiencies (ESID) and European reference network on rare primary immunodeficiency, autoinflammatory and autoimmune diseases (ERN RITA) complement guideline: deficiencies, diagnosis, and management. J Clin Immunol. 2020;40(4):576–591. doi: 10.1007/s10875-020-00754-1 32064578 PMC7253377

[54] Ali YM, Lynch NJ, Haleem KS, et al. The lectin pathway of complement activation is a critical component of the innate immune response to pneumococcal infection. PLoS Pathog. 2012;8(7):e1002793. doi: 10.1371/journal.ppat.1002793 22792067 PMC3390405

[55] Smith T, Cunningham-Rundles C. Primary B-cell immunodeficiencies. Hum Immunol. 2019;80(6):351–362. doi: 10.1016/j.humimm.2018.10.015 30359632 PMC7395616

[56] Stabler S, Giovannelli J, Launay D, et al. Serious infectious events and immunoglobulin replacement therapy in patients with autoimmune disease receiving rituximab: a retrospective cohort study. Clin Infect Dis. 2021;72(5):727–737. doi: 10.1093/cid/ciaa127 32067031

[57] Wudhikarn K, Palomba MR, Pennisi M, et al. Infection during the first year in patients treated with CD19 CAR T cells for diffuse large B cell lymphoma. Blood Cancer J. 2020;10(8):79. doi: 10.1038/s41408-020-00346-7 32759935 PMC7405315

[58] Brouwer S, Rivera-Hernandez T, Curren BF, et al. Pathogenesis, epidemiology and control of group A *Streptococcus* infection. Nature reviews. Nat Rev microbiol. 2023;21(7):431–447. doi: 10.1038/s41579-023-00865-7 36894668 PMC9998027

[59] Unoarumhi Y, Davis ML, Rowe LA, et al. A novel invasive *Streptococcus pyogenes* variant sublineage derived through recombinational replacement of the *emm*12 genomic region. Sci Rep. 2023;13(1):21510. doi: 10.1038/s41598-023-48035-2 38057343 PMC10700362

[60] Zhang W, Rong C, Chen C, et al. Type-IVC secretion system: a novel subclass of type IV secretion system (T4SS) common existing in Gram-positive genus *Streptococcus*. PLOS ONE. 2012;7(10):e46390. doi: 10.1371/journal.pone.0046390 23056296 PMC3464263

[61] Lukomski S, Bachert B, Squeglia F, et al. Collagen-like proteins of pathogenic streptococci. Mol microbiol. 2017;103(6):919–930. doi: 10.1111/mmi.13604 27997716 PMC5344740

[62] Oliver-Kozup HA, Elliott M, Bachert BA, et al. The streptococcal collagen-like protein-1 (Scl1) is a significant determinant for biofilm formation by group A *Streptococcus*. BMC microbiol. 2011;11(1):262. doi: 10.1186/1471-2180-11-262 22168784 PMC3268755

[63] Flores AR, Jewell BA, Versalovic EM, et al. Natural variant of collagen-like protein a in serotype M3 group A *Streptococcus* increases adherence and decreases invasive potential. Infect Immun. 2015;83(3):1122–1129. doi: 10.1128/iai.02860-14 25561712 PMC4333440

[64] Lynskey NN, Goulding D, Gierula M, et al. RocA truncation underpins hyper-encapsulation, carriage longevity and transmissibility of serotype M18 group A streptococci. PLoS Pathog. 2013;9(12):e1003842. doi: 10.1371/journal.ppat.1003842 24367267 PMC3868526

[65] Parks T, Wilson C, Curtis N, et al. Polyspecific intravenous immunoglobulin in clindamycin-treated patients with streptococcal toxic shock syndrome: a systematic review and meta-analysis. Clin Infect Dis. 2018;67(9):1434–1436. doi: 10.1093/cid/ciy401 29788397 PMC6186853

