# Supplementary material for: Recurrence following invasive GAS infections in adults: Triumph of virulence or failure of immunity?
Source: Virulence. 2025 Sep 29;16(1):2563765. doi: 10.1080/21505594.2025.2563765 (PMC12482427; doi:10.1080/21505594.2025.2563765)
Supplement: Supplementary figures and tables legends.docx [file KVIR_A_2563765_SM6083.docx]

Figure S1: Minimum spanning tree (wgMLST, Bionumerics v8.1) of Streptococcus pyogenes isolates from initial invasive infection (iGAS) and recurrence.

Figure S2: Schematic representation of the arrangement of genes in the Mga locus of GAS isolates found in patients A to D of different emm types. The sic gene was identified only in emm1 isolates, and the drs gene was found in emm12 isolates, except for the isolate of emm12 from Patient C´s recurrent iGAS episode.

Supplementary Table S1: Panel of genes associated with immune and hematopoietic disorders

Supplementary Table S2: Prevalence of virulence genes in the comparative genomic dataset based on predictions using the ABRicate tool with the VFDB database

Supplementary Table S3: Charlson comorbidity index

Supplementary Table S4: A summary of results of antimicrobial susceptibility testing and typing

Supplementary Table S5: Patient laboratory parameters

Supplementary Table S6: Overview of the antibiotic treatment in patients A-D

Supplementary Table S7: Detailed annotation of T4SS-type integrative conjugative element in GAS10 strain (patient A) predicted by ICEfinder with ICEberg 3.0 database

Supplementary Table S8: Detailed annotation of T4SS-type integrative conjugative element in GAS63 strain (patient C) predicted by ICEfinder with ICEberg 3.0 database

Supplementary Table S9: Prevalence of ICE-sssociated T4SS genes in the comparative genomic dataset

Supplementary Table S10: The results of a single nucleotide polymorphism analysis of GAS482 from patient C mapped to reference MGAS9429

Supplementary Table S11: The results of a single nucleotide polymorphism analysis of GAS226 from patient D mapped to reference MGAS9429

Supplementary Table S12: Prevalence of selected gene mutations in the comparative genomic dataset

Supplementary Table S13: Prevalence of SclA protein with deletion of 79-aa region in the comparative genomic dataset

Supplementary Table S14: Project accession numbers for external sequence data included in the comparative genomic dataset
